# Supplementary material for: Detailed molecular and epigenetic characterization of the pig IPEC-J2 and chicken SL-29 cell lines
Source: iScience. 2023 Feb 20;26(3):106252. doi: 10.1016/j.isci.2023.106252 (PMC10018572; doi:10.1016/j.isci.2023.106252)
Supplement: Data S2. Complete homer output for identified motifs in Chicken SL-29, related to Tables 5 and 6 — Homer motif analysis results for histone modifications H3K4me1, H3K4me3, H3K27ac, enhancers, and ATAC-seq of chicken SL-29 cell line. Parameters for possible false positives is as mentioned earlier for S5. [file mmc3.zip › Data_S2/S6/Chicken_SL_29/peak_files_CTCF/homerResults/motif3.similar.html]

motif3

## Information for motif3

A
G
T
C
A
C
T
G
A
G
T
C
A
G
T
C
G
T
A
C
A
G
T
C
A
G
T
C
A
C
G
T
T
A
C
G
A
T
C
G
A
G
C
T
A
C
T
G
A
C
T
G
  
Reverse Opposite:  

A
G
T
C
A
G
T
C
C
T
G
A
A
T
G
C
A
T
G
C
C
G
T
A
A
C
T
G
C
T
A
G
A
C
T
G
A
C
T
G
A
C
T
G
A
G
T
C
C
T
A
G
  

|  |  |
| --- | --- |
| p-value: | 1e-259 |
| log p-value: | -5.972e+02 |
| Information Content per bp: | 1.842 |
| Number of Target Sequences with motif | 1293.0 |
| Percentage of Target Sequences with motif | 4.39% |
| Number of Background Sequences with motif | 415.4 |
| Percentage of Background Sequences with motif | 1.43% |
| Average Position of motif in Targets | 151.8 +/- 73.0bp |
| Average Position of motif in Background | 153.0 +/- 95.3bp |
| Strand Bias (log2 ratio + to - strand density) | -0.1 |
| Multiplicity (# of sites on avg that occur together) | 1.08 |
| Motif File: | file (matrix) reverse opposite |

### Similar de novo motifs found

|  |  |  |  |  |  |  |  |
| --- | --- | --- | --- | --- | --- | --- | --- |
| Rank | Match Score | Redundant Motif | P-value | log P-value | % of Targets | % of Background | Motif file |
| 1 | 0.792 | A G T C C T G A A G T C A G T C C G T A A C T G C T G A A C G T A C T G A C T G | 1e-197 | -454.635482 | 3.12% | 0.97% | motif file (matrix) |
| 2 | 0.821 | T A C G A G T C G T A C T G C A A T G C A G C T T C G A T A C G T C G A C A T G C T A G A C T G T G A C | 1e-193 | -446.227328 | 3.02% | 0.93% | motif file (matrix) |
| 3 | 0.760 | C G T A C T A G C T G A A C T G C A T G A C T G T A G C C T A G | 1e-164 | -377.691474 | 14.42% | 9.44% | motif file (matrix) |
| 4 | 0.685 | G C A T C G T A T C A G C T A G A C T G A T C G A C T G G T A C C T A G A G T C | 1e-158 | -366.017745 | 4.77% | 2.14% | motif file (matrix) |
| 5 | 0.647 | A C T G A G T C G C A T A G T C A G T C G T A C G A C T A G T C A C G T G C T A A T C G G A C T A C T G | 1e-82 | -190.132299 | 0.30% | 0.02% | motif file (matrix) |
| 6 | 0.621 | C G T A T A G C G A C T C G T A A C T G C T A G A C G T C A T G C T A G A G T C C G T A G A T C C A G T | 1e-58 | -133.688594 | 0.23% | 0.02% | motif file (matrix) |
